# Ovarian function and X chromosome tissue mosaicism in adolescents with Turner syndrome and ongoing spontaneous puberty

**DOI:** 10.3389/fendo.2026.1866671

**Published:** 2026-07-07

**Authors:** Anna Turchinets, Olga Stupko, Elena Uvarova, Polina Tsabai, Alina Badlaeva, Alexandra Asaturova, Nail Kamaletdinov, Elena Khashchenko, Zaira Kumykova, Dmitry Trofimov, Svetlana Yureneva, Alla Gavisova, Gennady Sukhikh

**Affiliations:** 1 National Medical Research Center for Obstetrics, Gynecology and Perinatology Named after Academician V.I. Kulakov of the Ministry of Health of the Russian Federation, Moscow, Russia; 2 I.M. Sechenov First Moscow State Medical University of the Ministry of Health of the Russian Federation (Sechenov University), Moscow, Russia; 3 Pirogov Russian National Research Medical University of the Ministry of Health of the Russian Federation, Moscow, Russia

**Keywords:** adolescents, biobank, fertility preservation, follicle density, mosaicism, ovarian reserve, ovarian tissue cryopreservation, Turner syndrome

## Abstract

**Introduction:**

The maintenance of normal ovarian function depends on the presence of two structurally intact X chromosomes, which explains the high risk of premature ovarian insufficiency in individuals with Turner syndrome (TS). Most aneuploid germ cells undergo apoptosis during embryonic gonadal development, resulting in the formation of streak gonads in the majority of individuals with a 45,X karyotype. However, the role of X-monosomy in ovarian somatic cells, which provide the microenvironment essential for oocyte survival and development, remains poorly understood. To our knowledge, this is the first study to investigate the association between X chromosome aneuploidy in ovarian somatic cells and peripheral blood lymphocytes and ovarian function in adolescents with mosaic TS. The aim of this exploratory case-series study was to comprehensively evaluate the clinical, hormonal, ultrasonographic, morphological, morphometric, immunohistochemical, and molecular cytogenetic characteristics of the ovaries in adolescents with mosaic TS and spontaneous pubertal development.

**Methods:**

Five adolescents with mosaic TS underwent assessment of ovarian reserve markers and ovarian tissue cryopreservation. Follicle density (FD), expression of oocyte-specific markers (GDF9, BMP15, and CD117), and X chromosome mosaicism in 2–6 oocytes, 50–300 granulosa cells (GCs), and 50 ovarian stromal cells per participant were evaluated.

**Results:**

Despite ongoing puberty and normal gonadotropin levels, reduced serum anti-Müllerian hormone (AMH) and inhibin B concentrations were observed in some participants. A normal X chromosome complement was identified in all analyzed oocytes. Ovarian stromal cells were predominantly monosomic for the X chromosome, whereas GCs demonstrated substantial intra- and inter-individual variability in X chromosome mosaicism. No clear relationship was observed between X chromosome mosaicism in peripheral blood lymphocytes and ovarian tissue. Age-appropriate FD was observed only in participants whose follicles contained more than 60% non-monosomic GCs (XX or XXX).

**Discussion:**

The findings suggest that ovarian function in adolescents with mosaic TS may be associated not only with the chromosomal status of oocytes but also with that of GCs. Peripheral blood karyotype did not reliably reflect ovarian tissue mosaicism in this exploratory series, highlighting the importance of comprehensive evaluation of ovarian reserve markers when counseling patients with TS regarding fertility preservation.

## Introduction

Turner syndrome (TS) is a chromosomal disorder caused by complete or partial monosomy or by structural abnormalities of the X chromosome. The diagnosis is typically established by karyotype analysis of peripheral blood lymphocytes (1). TS affects nearly 1 in 2,500 live-born females (2). Delayed pubertal development and primary or secondary amenorrhea are among the most common clinical manifestations of TS and occur in approximately 80-90% of affected adolescents (3, 4). These features are largely attributable to premature ovarian insufficiency (POI), a condition that is relatively uncommon during adolescence but for which TS accounts for nearly half of all cases (5, 6).

The relationship between X chromosome abnormalities and ovarian dysfunction has been extensively investigated. Studies of ovarian tissue from fetuses with 45,X karyotype have demonstrated that primordial germ cell migration to the developing gonads is preserved during embryogenesis. However, folliculogenesis fails to initiate despite the presence of oogonia at various stages of fetal development (7). It has therefore been proposed that the absence of a second X chromosome disrupts chromosomal synapsis during the diplotene stage of meiotic prophase I, leading to accelerated germ-cell loss through apoptosis. Consequently, most individuals with a 45,X karyotype are born with severely depleted ovarian follicle pools, whereas patients with mosaic TS generally exhibit reduced follicle density (FD) compared with age-matched healthy females (8–10). In addition to germ-cell loss, the X chromosome abnormalities in ovarian somatic cells, including granulosa and stromal cells, may also be associated with altered follicle development (11–13).

Despite the high risk of ovarian insufficiency, approximately 10-20% of adolescents with TS experience spontaneous menarche and menstrual cycles (14–16). Furthermore, nearly half of these individuals, most commonly those with a mosaic 45,X/46,XX karyotype, retain ovarian function into adulthood and may achieve spontaneous pregnancies and live births (4, 17, 18). Spontaneous fertility has also been reported, albeit rarely, in women with non-mosaic 45,X TS (17, 19). The considerable variability in ovarian function observed among individuals with similar peripheral blood karyotypes suggests that tissue-specific X chromosome mosaicism within the ovary may contribute to differences in reproductive potential.

Because ovarian reserve (OR) declines unpredictably in TS, fertility preservation strategies such as ovarian tissue cryopreservation (OTC) are increasingly being considered (1). Previous studies have demonstrated that the majority of immature oocytes recovered from ovarian cortical tissue of patients with TS contain a normal complement of X chromosomal sister chromatids during meiotic prophase I (20–22). Moreover, despite the high incidence of X-monosomy in granulosa and stromal cells, primordial follicles in these patients retain the capacity to develop into secondary and even antral follicles (23, 24).

Given the marked variability of ovarian function among patients with similar cytogenetic forms of TS, further investigation of the ovarian follicular pool and X chromosome mosaicism in oocytes, GCs and ovarian stromal cells is warranted. Therefore, the aim of this study was to evaluate the morphological, immunohistochemical, and molecular cytogenetic characteristics of ovarian tissue in adolescents with mosaic TS and evidence of ongoing ovarian function, and to explore the relationship between ovarian-cell X chromosome mosaicism and markers of OR.

## Materials and methods

### Patients

This exploratory translational case-series study included five participants aged 11–15 years with TS and no evidence of Y chromosome material. All participants had undergone spontaneous pubertal development and were followed at the 2^nd^ Gynecology Department of the National Medical Research Center for Obstetrics, Gynecology and Perinatology Named after Academician V.I. Kulakov. Mosaic TS was confirmed in all participants by conventional karyotyping of peripheral blood lymphocytes, with analysis of at least 30 metaphases.

Clinical, laboratory, and instrumental assessments were performed to evaluate ovarian function. Sexual maturation was assessed according to Tanner staging (25). After laboratory and ultrasonographic confirmation of preserved OR (follicle-stimulating hormone (FSH) <25 IU/L and anti-Müllerian hormone (AMH) >0.01 ng/mL, and the presence of antral follicles by ultrasound examination), patients and their legal representatives were counseled regarding the risks of POI and available fertility-preservation options.

**Figure 1:**
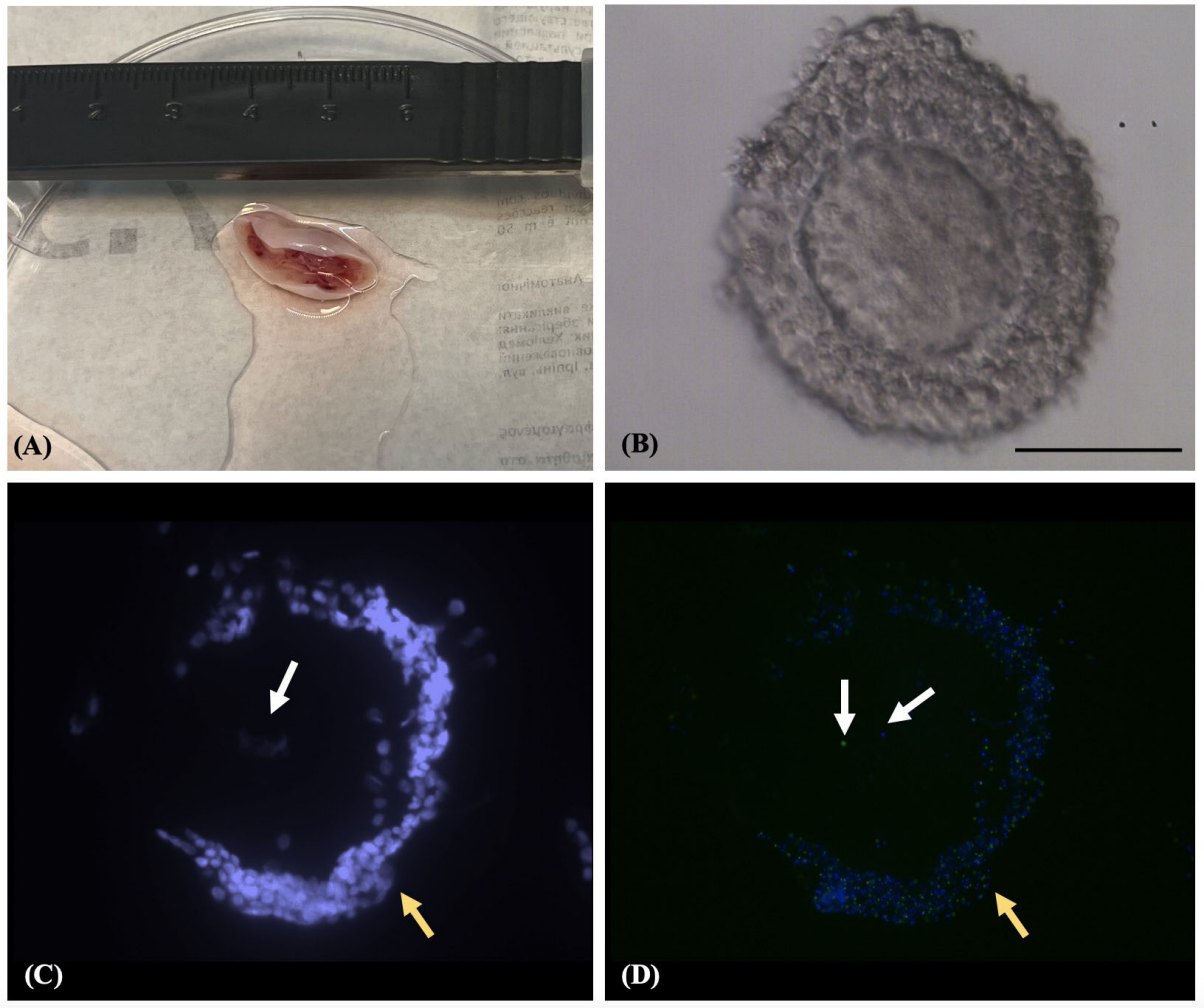
Isolation of ovarian follicles during tissue processing and fluorescence *in situ* hybridization (FISH) analysis. **(A)** Macroscopic appearance of an ovarian tissue fragment before cryopreservation. **(B)** Light microscopy image of a small follicle isolated during ovarian tissue processing (200× magnification). **(C)** DAPI (4′,6-diamidino-2-phenylindole) staining of an oocyte (white arrow), and granulosa cells (GCs; yellow arrow) (200× magnification). **(D)** FISH signals corresponding to centromeric regions of chromosomes 18 (blue) and X (green) in an oocyte (white arrows) and GCs (yellow arrow) (200× magnification). Scale bar: 100 μm.

Following written informed consent from participants or their legal representatives, all participants underwent laparoscopic unilateral partial oophorectomy for OTC. Immediately after resection, the fragment of ovary was placed in a sterile container filled with prewarmed Ooklin IVF medium (PanEco, Russia) and transported to the embryology laboratory in a temperature-controlled container for further processing. Ovarian tissue fragments measuring no more than 2.5x2.0x1.0 mm were subjected to comprehensive morphometric, morphological, and molecular cytogenetic analyses. During tissue dissection, the cortical and medullary layers were separated, and 2–6 small follicles (late primary and early secondary follicles) were isolated from each participant for molecular cytogenetic analysis using fluorescence *in situ* hybridization (FISH) (**Figures 1A–D**).

High-resolution images of microscopic specimens were acquired using an Axio Imager A2 microscope (Carl Zeiss, Germany) equipped with a CoolCube1 camera and ISIS software (MetaSystems, Germany) were used.

OTC was performed according to a standard slow freezing protocol (26).

### Ethics approval

The study was conducted in accordance with the World Medical Association International Code of Medical Ethics (Declaration of Helsinki). The protocol was approved by the Ethics Committee for Biomedical Research of the National Medical Research Center for Obstetrics, Gynecology and Perinatology named after Academician V.I. Kulakov of the Ministry of Health of the Russian Federation (Protocol № 12, December 1, 2022). Written informed consent for participation and the use of clinical data for research purposes was obtained from all participants and their legal representatives in cases where the individuals were under the age of 15.

### Hormonal assessment

Serum hormone concentrations were measured on days 3–5 of the menstrual cycle in participants with spontaneous menstruation. Serum levels of FSH, luteinizing hormone (LH), estradiol, testosterone, AMH, thyroid-stimulating hormone (TSH), free thyroxine (FT4), and prolactin were determined using electrochemiluminescence immunoassays on a Cobas e 411 analyzer (Roche, Switzerland). Serum inhibin B concentrations were measured using enzyme immunoassay, the Inhibin B Gen II ELISA kit (Beckman Coulter, USA), and absorbance was recorded using an Infinite F50 microplate reader (Tecan, Switzerland). Venous blood samples were collected into EDTA-containing tubes and stored in the laboratory for collection and storage of biomaterial samples of the National Medical Research Center for Obstetrics, Gynecology and Perinatology named after Academician V.I. Kulakov.

### Ultrasound examination

Transabdominal ultrasonography was performed using a MyLabClass C ultrasound system (Esaote, Italy) equipped with linear and convex transducer operating at frequencies of 1.8-6.0 MHz. In menstruating participants, examinations were performed on days 5–7 of spontaneous menstrual cycle. Ovarian and uterine volumes were calculated using the ellipsoid formula: Volume = length × width × anteroposterior diameter × 0.523.

Antral follicle count (AFC) was determined by counting all follicles measuring more than 2 mm in diameter throughout the ovary.

### Histological analysis

Formalin-fixed, paraffin-embedded ovarian tissue samples were processed routinely and stained with hematoxylin and eosin. Digital images were acquired using a Leica Aperio AT2 slide scanner (Leica Biosystems, Germany). To determine tissue area and estimate the number of primordial follicles, every fifth 10-μm section was analyzed using QuPath software (version 0.3.2). FD was calculated according to the method described by Schmidt et al. (27). To avoid double-counting of follicles, a modified correction factor proposed by Hassan et al. was applied (9). Detailed formulas used for FD calculation are provided in the Supplementary Materials and Methods.

Follicles were classified according to the criteria described by Gougeon et al. and Williams et al. (28, 29). Follicular quality was assessed using the morphological criteria proposed by Gougeon (30).

### Immunohistochemistry

Paraffin sections (4 μm thick) were mounted on positively charged slides. After deparaffinization in xylene and graded ethanol solutions (96%, 85%, and 70%), the sections were rinsed in distilled water. Three antibodies were used to assess the expression of proteins and growth factors involved in preantral folliculogenesis and oocyte maturation: GDF9 (growth differentiation factor 9; polyclonal; Cusabio, USA), BMP15 (bone morphogenetic protein 15; polyclonal; Cusabio, USA), CD117 (c-Kit, receptor tyrosine kinase; clone EP10; Ventana, Roche, Switzerland). Immunostaining was performed using the BenchMark XT automated staining platform (Ventana, Roche, Switzerland) according to the manufacturer’s protocols. Antibody incubation was performed at 37 °C for 16–24 minutes. Immunoreactivity was visualized using the DAB Universal Detection Kit (Ventana, Roche, Switzerland). GDF9 and BMP15 antibodies were used at dilutions of 1:400 and 1:25, respectively, whereas CD117 was used according to the manufacturer’s recommendations. Immunohistochemical staining intensity was evaluated using a semi-quantitative immunoreactive index (I-score): I-score = A × B, where A represents the proportion of positively stained cells and B represents staining intensity (31). Scores of 0-2, 3-4, 5-8, and 9–12 corresponded to negative, weak, moderate, and strong expression, respectively.

### Fluorescence *in situ* hybridization

Commercially available centromeric DNA probes for chromosomes X (DXZ1), Y (DYZ3), and 18 (D18Z1) (Kreatech Biotechnology, Netherlands) were used. VECTASHIELD mounting medium containing DAPI (4′,6-diamidino-2-phenylindole fluorescent DNA stain) (Vector Laboratories, USA) was applied for nuclear counterstaining.

To assess aneuploidy in oocytes and GCs, isolated follicles were placed on individual microscope slides in 100 μl droplets of 0.04 M KCl and incubated at 37 °C for 20 minutes. Slides were air-dried and pre-fixed with 300 μl of 0.05 M KCl for 2 minutes. The fixative consisted of absolute ethanol and glacial acetic acid mixed in a 3:1 ratio. After fixation, 4 µl of probe mixture was applied to each slide. Samples were denatured at 82 °C for 7 minutes and hybridized at 37 °C for 16–22 hours using the ThermoBrite system (StatSpin, USA). Post-hybridization washes were performed using 2× SSC and 0.4× SSC buffers, followed by dehydration in graded ethanol solutions (70%, 85%, and 99%). Finally, 5 µl of DAPI-containing mounting medium was applied.

For FISH analysis of ovarian stromal cells, 4-µm paraffin sections of ovarian tissue were mounted on poly-L-lysine-coated slides, deparaffinized, and rehydrated. Slides were incubated in 2% EDTA at 90 °C for 25 minutes and subsequently treated with a pepsin solution containing 0.4% hydrochloric acid and 5% pepsin in a 9:1 ratio. After incubation for 50 minutes at 37 °C, slides were washed using 2× SSC buffer, dehydrated through graded ethanol solutions (70%, 85%, and 96%), and hybridized at 75 °C for 5 minutes and at 37 °C for 18 hours. Post-hybridization washes were performed using 2× SSC buffer twice, followed by dehydration describes earlier.

X chromosome centromeric signals (green) were evaluated only in cells displaying two chromosome 18 centromeric signals (blue). 50 GCs from each follicle and in 50 stromal cells from each ovarian tissue sample were analyzed, primarily in peripheral regions of the tissue sections.

Given the very small sample size and non-normal data distribution, all statistical analyses were considered purely exploratory. Statistical analyses were performed using Jamovi version 2.5.7.0 (Australia) and GraphPad Prism version 9.3.1 (USA). Continuous variables were summarized as medians and interquartile ranges (IQRs). Group comparisons were performed using the Mann-Whitney U-test. Cliff’s delta (δ) was calculated as a measure of effect size and interpreted as small (≥0.11<0.28), medium (≥0.28<0.43), and large (≥0.43), according to Vargha and Delaney (32, 33). Spearman correlation coefficients were calculated for descriptive purposes only and were not used for inferential interpretation.

## Results

### Clinical, hormonal and ultrasonographic assessment

As shown in **Table 1**, the ages at spontaneous thelarche and menarche did not differ substantially from population-based reference values for Russian adolescent females (34, 35). Among the four participants who had experienced menarche (P1, P2, P4, and P5) three (P1, P2, and P4) had irregular menstrual cycles, including oligomenorrhea and abnormal uterine bleeding. Assessment of menstrual function in participant P5 was not informative because less than three months had elapsed since menarche. To characterize the hormonal and ultrasonographic parameters of the study cohort, values were compared with validated reference data for healthy Russian adolescent females at Tanner stages B3-B4 Tanner reported by Khashchenko (36). No difference was observed for prolactin, estradiol, and testosterone concentrations (δ<0.11). Median serum concentrations of LH and TSH in patients with TS were within the expected reference range, and effect sizes were small-to-medium (δ<0.28). Large effect sizes were observed for FSH and FT4 levels, and uterine volume despite the absence of statistically significant differences, likely reflecting limited statistical power associated with the very small sample size (δ=0.48; δ=0.52, respectively). AMH levels, ovarian volumes, and AFC were lower than those reported in healthy adolescents, with the large effect sizes observed for all comparisons (δ>0.43). Given the exploratory nature of the study, these findings should be interpreted descriptively. Serum AMH concentrations were below 1.1 ng/mL in three participants (P1, P4, P5), whereas inhibin B concentrations were below 10 pg/mL in two participants (P1, P5).

**Table 1:** Clinical, hormonal and ultrasonographic characteristics of the study participants and comparison with Tanner stage-matched reference values from healthy Russian adolescent females.

Parameters	Patient 1	Patient 2	Patient 3	Patient 4	Patient 5	Median participants’ values; IQRs	Median normative values; IQRs (5th-95th percentile)*	P-value**	δ***
Karyotype	45,X(91%) /46,XX(9%)	45,X(67%) /46,XX(33%)	45,X(81%) /47,XXX(19%)	45,X(55%) /46,XX(45%)	45,X(13%) /46,XX(87%)	–	–	–	–
Age, years	15	12	11	15	11	12; 11-15	–	–	–
Age of telarche, years	10	8	10	12	9	10; 8.5-11.0	–	–	–
Age of menarche, years	12	10	–	14	11	11.5; 10.3-13.5	–	–	–
Tanner stage	B4	B3	B3	B4	B3	–	–	–	–
Menstrual cycle	O	O, AUB	–	AUB	–	–	–	–	–
FSH, IU/L	3.1	3.7	5.1	3.0	4.7	3.7; 3.1-4.9	5.5; 4.1-6.5 (2.7-8.2)	0.090	0.48
LH, IU/L	N/A	7.0	2.0	4.5	8.2	5.8; 6.2-7.9	3.9; 2.5-4.9 (2.0-8.0)	0.452	-0.24
Prolactin, mIU/L	508.4	134.3	327.6	164.5	154.2	164.5; 144.3-418.0	191.0; 146.0-288.0 (102.0-490.0)	0.851	-0.06
TSH, mIU/L	3.9	1.8	1.8	0.8	2.3	1.8; 1.3-3.1	1.9; 1.5-2.2 (1.1-2.7)	0.706	-0.11
FT4, pmol/L	13.8	14.7	17.8	19.6	14.9	14.9; 14.3-18.7	13.9; 13.0-14.9 (12.0-17.5)	0.063	-0.58
Estradiol, pmol/L	214.9	142.4	203.7	331	<18.4	203.7; 80.4-273.0	179.0; 117.0-210.0 (75.1-338.0)	0.777	-0.09
Testosterone, nmol/L	1.1	1.2	0.9	0.8	0.5	0.9; 0.7-1.2	0.8; 0.7-1.0 (0.5-1.4)	0.783	-0.08
AMH, ng/mL	0.99	5.22	5.25	0.96	0.60	0.99; 0.78-5.24	5.80; 3.9-6.9 (2.15-7.59)	0.025	0.64
Inhibin B, pg/mL	9.2	69.1	144.7	41.4	9.9	41.4; 9.6-106.9	N/A	–	–
Uterine volume, cm3	22.4	26.8	8.9	15.2	24.5	22.4; 12.1-25.7	27.3; 22.2-36.9 (19.3-52.1)	0.066	0.52
Right ovary volume, cm3	5.9	6.4	4.8	3.1	4.1	4.8; 3.6-6.2	8.2; 6.0-10.2 (3.8-14.9)	0.012	0.69
Left ovary volume, cm3	2.2	2.8	3.8	2.8	0.8	2.8; 1.5-3.3	7.8; 5.2-10.9 (2.8-13.1)	0.002	0.89
AFC of right ovary, number	4	5	10	5	4	5; 4-8	8; 8-10 (6-13)	0.013	0.72
AFC of left ovary, number	3	4	8	4	3	4; 3-6	8; 7-9 (6-12)	0.005	0.80

NA, not available or not applicable; O, oligomenorrhea; AUB, abnormal uterine bleeding;.

FSH, follicle-stimulating hormone; LH, luteinizing hormone; TSH, thyroid stimulating hormone; FT4, free thyroxine; E2, estradiol; AMH, anti-Müllerian hormone; AFC, antral follicle count; IQR, interquartile range.

*Reference hormonal and ultrasonographic values for healthy Russian adolescent females at Tanner stages B3-B4 according to Khashchenko (36).

**p-values were calculated using the Mann–Whitney U-test.

***δ, Cliff’s delta.

### Histological and immunohistochemical analysis

Comparison of FD values (**Table 2**) with age-normalized reference data for European adolescent females reported by Hassan et al., demonstrated that four patients (P1-P4), who also had the highest AMH concentrations, exhibited FD values within or above the expected age-specific range (-2 SD to + 2SD) (9). In contrast, participant P5, who had the lowest AMH concentration, demonstrated an FD value below the lower limit of the age-specific reference range (84–390 n/mm^3^) (9).

**Table 2:** Morphological characteristics of ovarian follicles and follicle density (FD) in the study participants.

Parameters	Patient 1	Patient 2	Patient 3	Patient 4	Patient 5
Karyotype	45,X(91%) /46,XX(9%)	45,X(67%) /46,XX(33%)	45,X(81%) /47,XXX(19%)	45,X(55%) /46,XX(45%)	45,X(13%) /46,XX(87%)
FD, n/mm3 (age-normalized reference values*)	26 (25-203)	101 (84-390)	250 (84-390)	145 (25-203)	24 (84-390)
Follicle stages	primordial and primary	primordial, primary and secondary, antral	primordial, primary, secondary, antral	primordial and primary	primordial and primary
Pale nuclear material	+	–	+	–	+
Incomplete layer of GCs	+	–	+	+	+
Dioocytic follicles	–	–	+	–	–
Empty follicles	+	+	–	+	–
Swelling of GCs	+	–	–	–	+
Contracted ooplasm	+	–	+	+	–
Karyopyknosis of GCs	–	–	–	–	+
Karyopyknosis of oocyte	–	–	+	+	–
Follicles with normal morphology	+	+	+	+	+

FD, follicle density; GCs, granulosa cells;.

*age-specific reference values for FD according to Hassan et al. (9).

**Figure 2:**
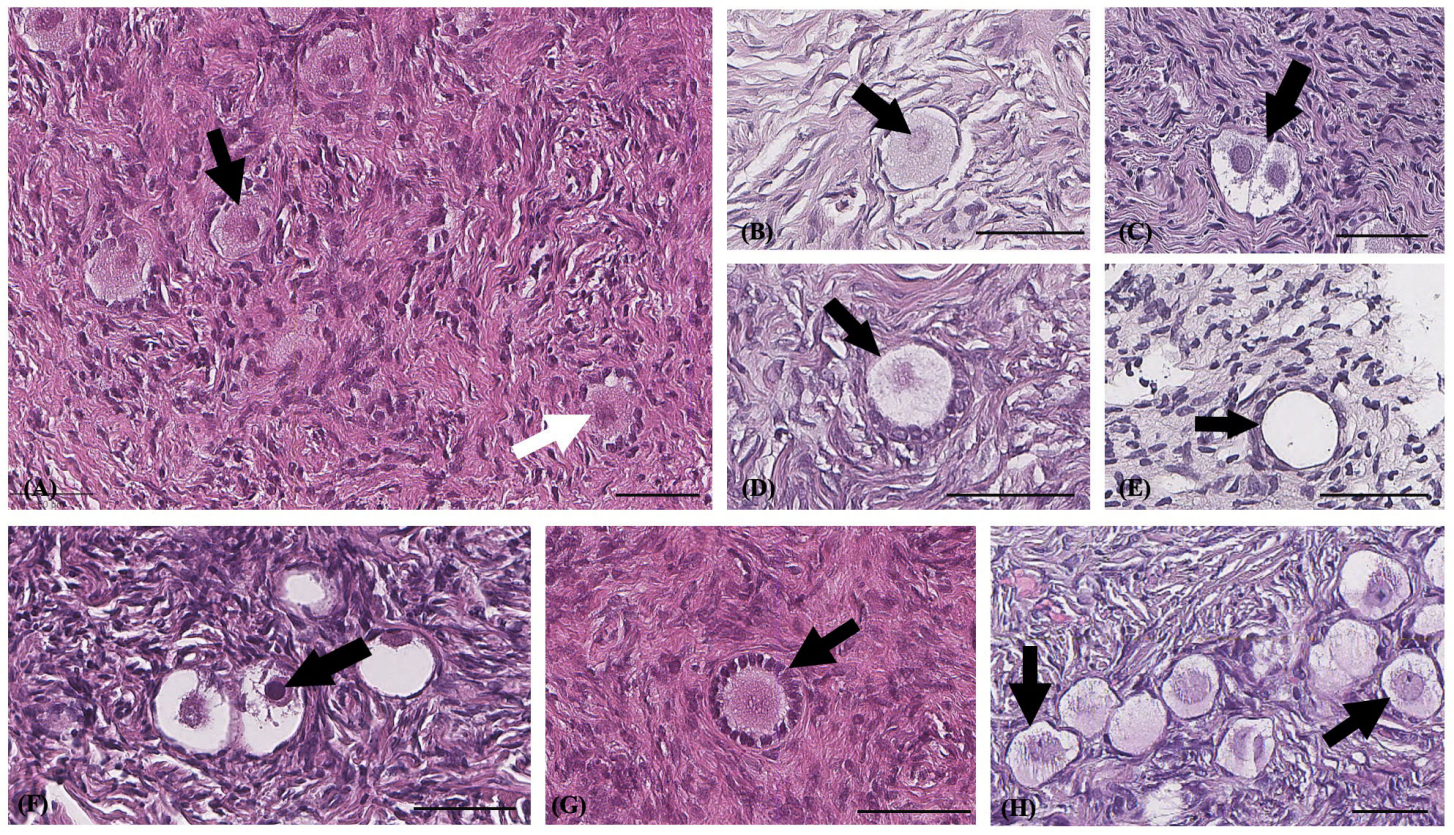
Histological analysis of ovarian biopsy samples from patients with Turner syndrome (TS). **(A)** Primordial (black arrow) and primary follicles (white arrow) with normal morphology. **(B–H)** Follicles with abnormal morphology: **(B)** pale oocyte nucleus; **(C)** dioocytic follicle; **(D)** incomplete granulosa-cell layer; **(E)** empty follicle; **(F)** karyopyknotic oocyte; **(G)** karyopyknotic granulosa cells (GCs); **(H)** contracted ooplasm. Scale bars: 50 μm.

Primordial follicles with abnormal morphology were identified in all ovarian tissue samples examined. The most common morphological abnormalities included an irregular layer of flattened GCs, a pale oocyte nucleus, absence of a discernible germinal vesicle, and retraction of oocyte cytoplasm (**Figures 2B–H**). These findings are consistent with degenerative follicular changes. Nevertheless, morphologically normal follicles at different developmental stages were observed in all participants, indicating preservation of follicular growth potential in at least a subset of follicles (**Figure 2A**).

**Figure 3:**
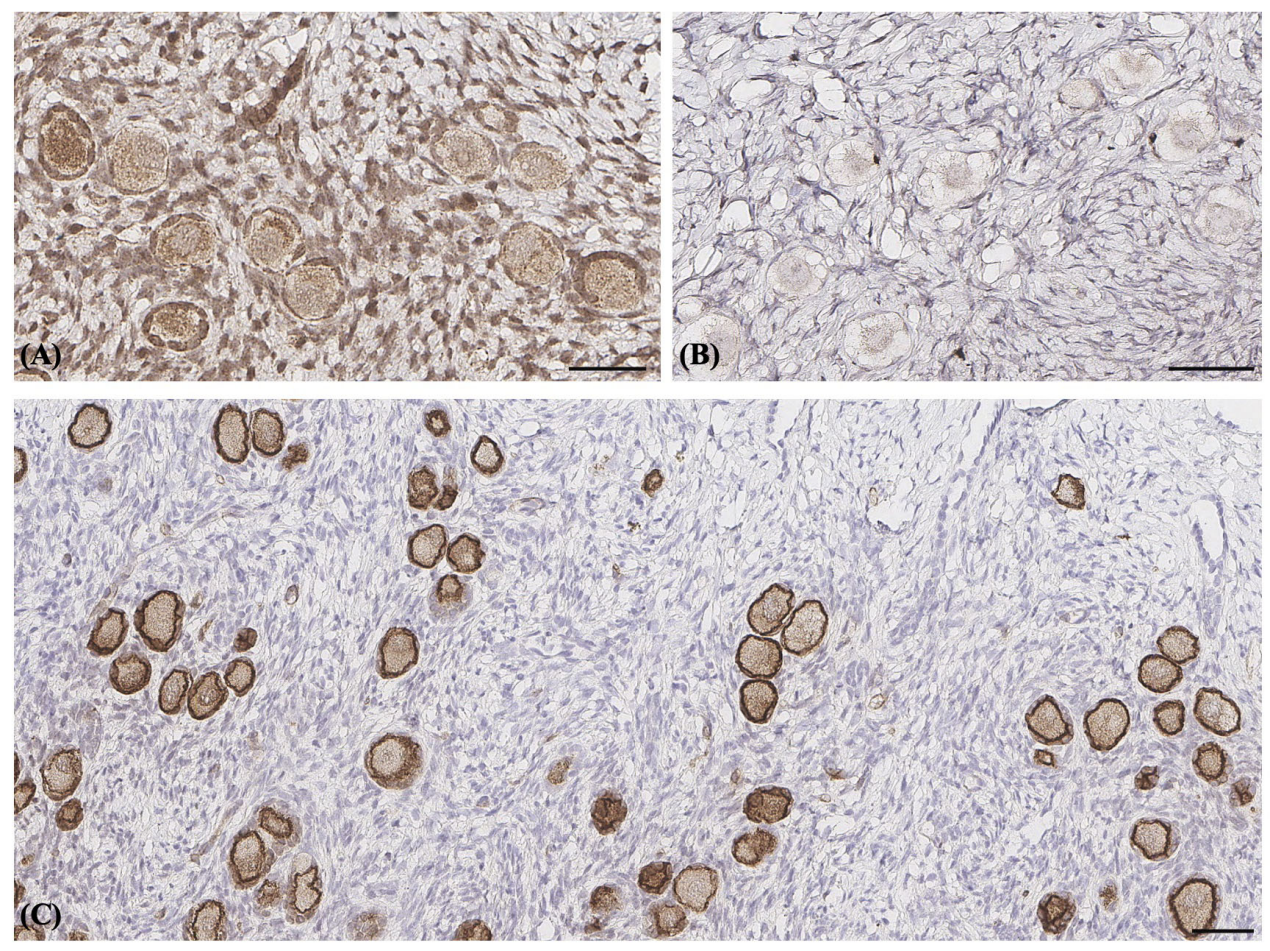
Immunohistochemical analysis of ovarian biopsy samples from patients with Turner syndrome (TS). **(A)** Moderate GDF9 expression in the ooplasm and granulosa cells (GCs) of primordial follicles (I-score = 6). **(B)** Weak BMP15 expression in the ooplasm of primordial follicles (I-score = 4). **(C)** Strong CD117 expression in the oolemma and ooplasm of primordial follicles (I-score = 9). Scale bars: **(A)** 20 μm, **(B, C)** 50 μm.

Expression of all evaluated oocyte-specific proteins and growth factors were detected in ovarian tissue samples from four patients (P1 and P3-P5). Moderate GDF9 expression (I-score – 6–8 points) and a weak reaction for BMP15 (I-score – 3–4 points) were observed in oocyte cytoplasm from these samples (**Figures 3A, B**). In participant P2, GDF9 expression in the ooplasm was weak (I-score – 4 points), whereas BMP15 expression was negative (I-score – 2 points), primarily because of the low proportion of positively stained cells (A-criteria). In contrast, strong staining for CD117 was observed on the oolemma and within the ooplasm (I-score – 9–12 points), and moderate GDF9 expression was detected in GCs (I-score – 6–8 points) across all analyzed samples (**Figure 3C**).

### FISH analysis of ovarian cells

**Figure 4:**
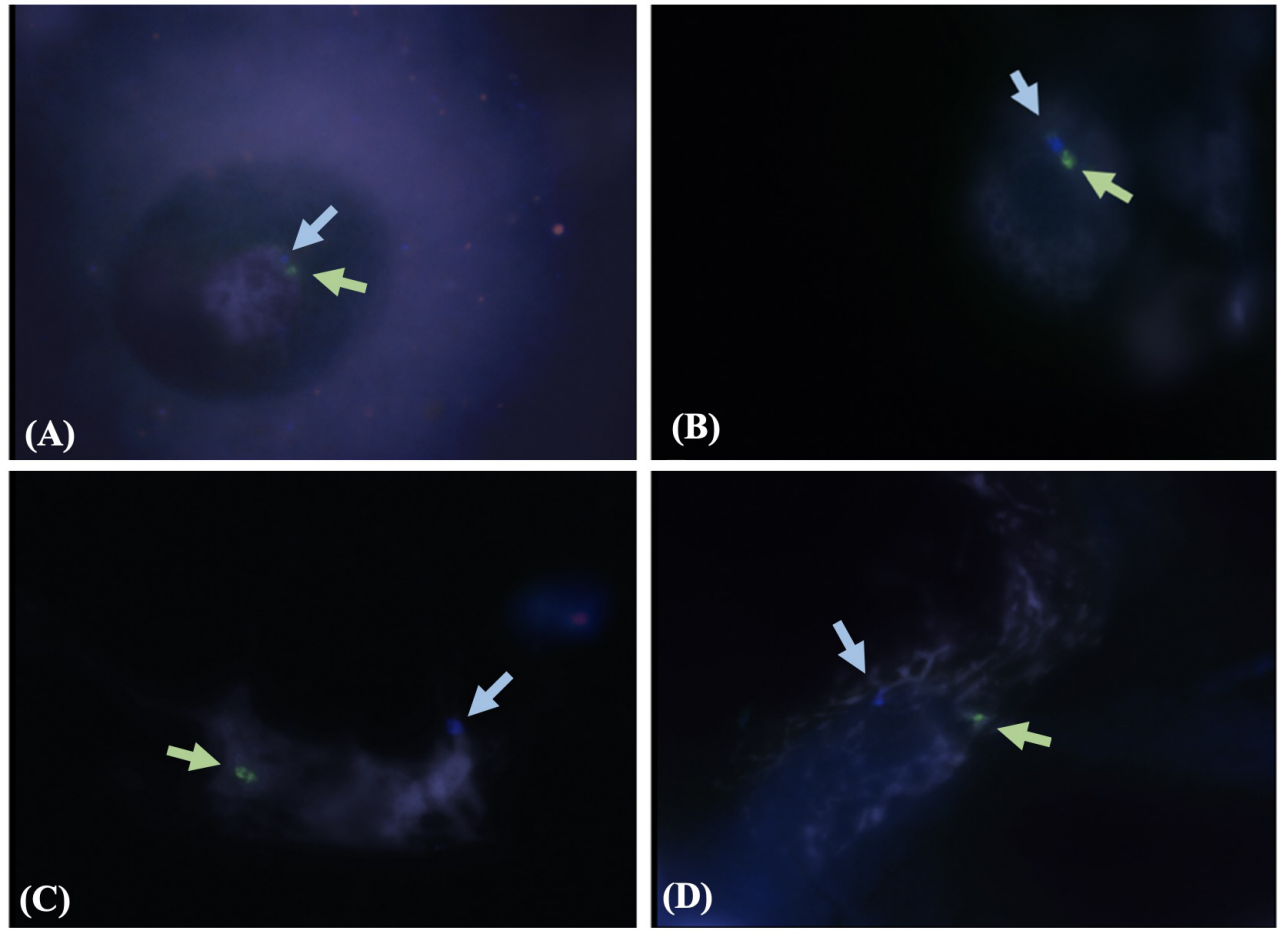
Fluorescence *in situ* hybridization (FISH) analysis of oocytes from patients with Turner syndrome (TS). **(A–D)** Single FISH signals corresponding to four chromatids of homologous X chromosomes (green arrows) and the control chromosomes 18 (blue arrows) in immature oocytes from different participants. In all cases, the signal area of X chromosome was comparable to that of chromosome 18 (1000× magnification).

Molecular cytogenetic analysis revealed no Y chromosome signals in any examined samples. Only a single signal for X chromosome and chromosome 18 was identified in all analyzed oocytes (**Figures 4A–D**). Because oocytes within primary and secondary follicles are arrested in prophase I of meiosis and contain four chromatids for each homologous chromosome pair (2n4c), paired chromatids are visualized as a single signal due to their close association within the synaptonemal complex (20). Signal intensity varied according to fluorophore characteristics, with chromosome 18 signals generally appearing weaker than X chromosome signals. In some cells, diffuse or non-compact signals were observed, limiting precise signal-area measurements and precluding reliable assessment of oocyte ploidy using FISH alone (37). Nevertheless, as shown in **Figures 4A–D**, the X chromosome signal was not appreciably smaller than the chromosome 18 control signal in any analyzed oocyte. These findings are consistent with previous observations suggesting the absence of X chromosome aneuploidy in oocytes from individuals with mosaic TS (20).

**Figure 5:**
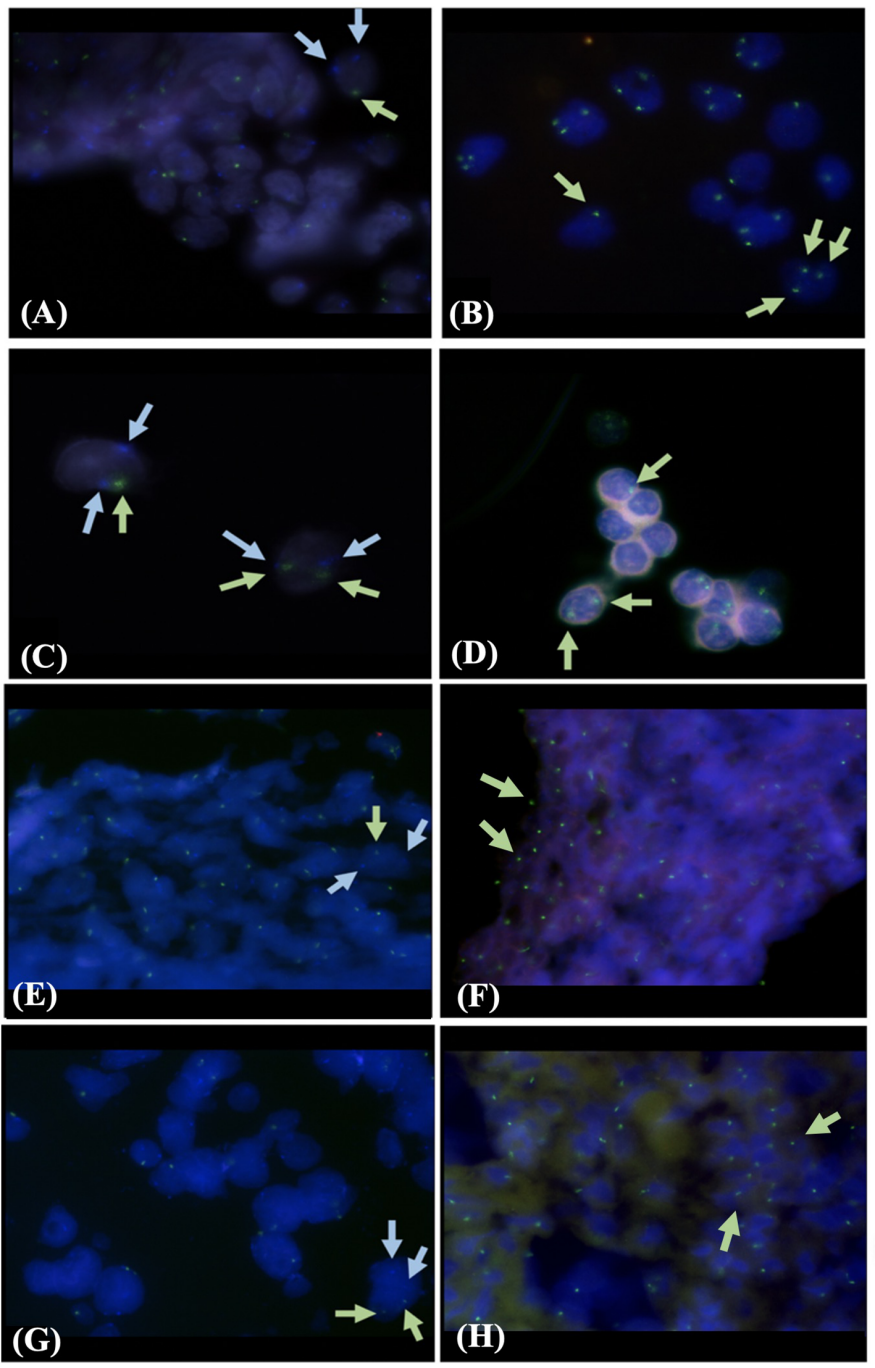
Fluorescence *in situ* hybridization (FISH) analysis of ovarian somatic cells from patients with Turner syndrome (TS). Green arrows indicate centromeric signals of X chromosome; blue arrows indicate centromeric signals of the chromosome 18. **(A–D)** X chromosome mosaicism in granulosa cells (GCs), with two chromosome 18 signals (1000× magnification). **(E–H)** Predominance of X chromosome aneuploidy in ovarian stromal cells (1000× magnification).

**Figure 6:**
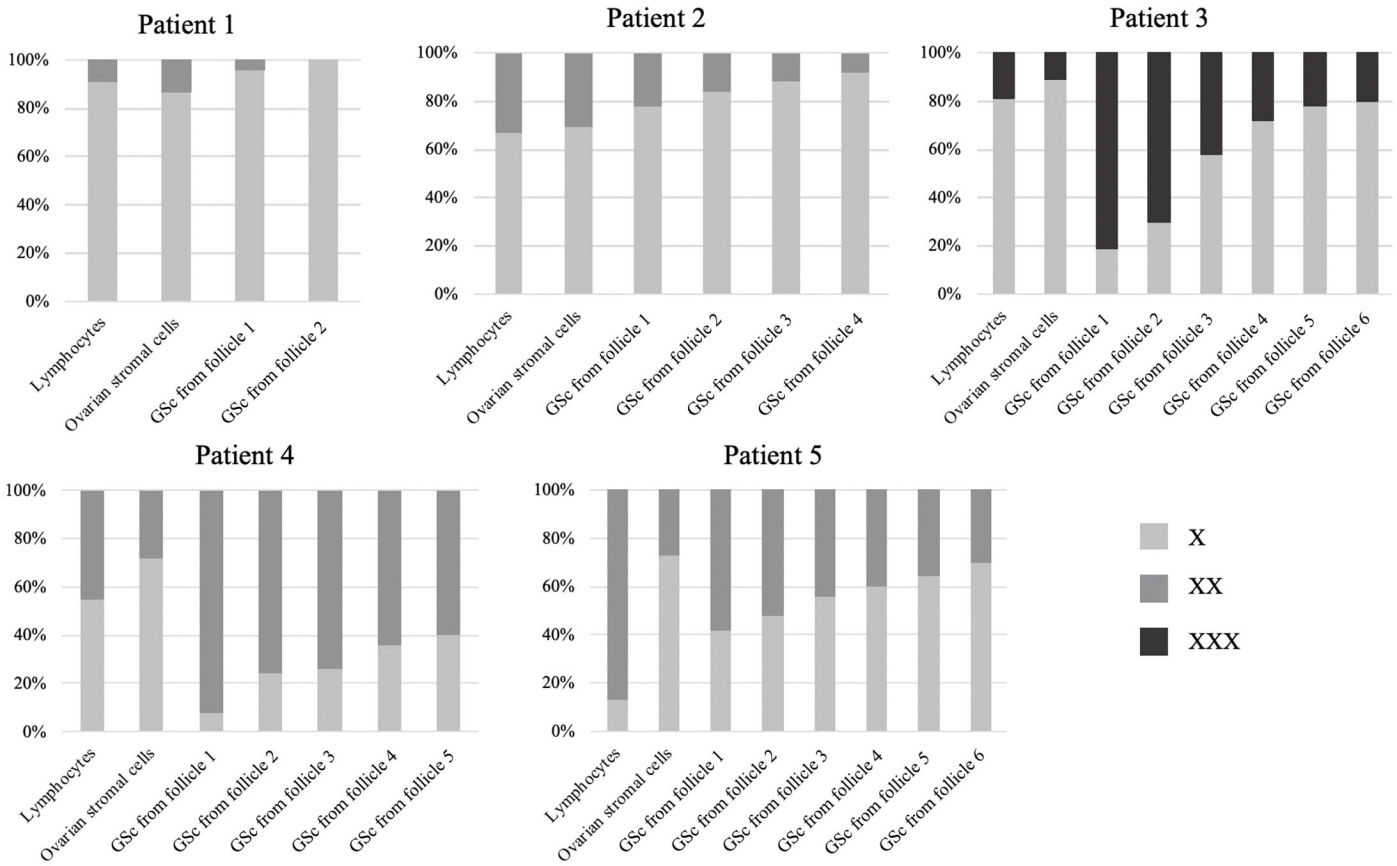
X chromosome mosaicism profiles in somatic cells from patients with Turner syndrome (TS). Peripheral blood lymphocytes (n=30-100), ovarian stromal cells (n=50), granulosa cells (GCs) from individual follicles (n=50) were analyzed. Variation in the proportion of X-monosomic cells was observed both between peripheral blood lymphocytes and GCs in individuals, and among GCs from different follicles within the same patient.

In three patients (P1, P2, and P5), the proportion of X-aneuploid GCs exceeded that observed in peripheral blood lymphocytes (**Figures 5A, C**, **6**). In contrast, participants P3 and P4 demonstrated lower rates of X-aneuploidy in GCs than in peripheral blood lymphocytes across all examined follicles. (**Figure 5B, D**, **6**). Notably, patients P3 and P4 also exhibited FD values above age-adjusted reference means reported by Hassan et al. (9). In contrast, participants P1, P2, and P5, in whom the proportion of X-aneuploid GCs exceeded that observed in peripheral blood lymphocytes, demonstrated FD values below -1 SD or even -2 SD relative to reference values. As shown in **Figure 6**, only patients with a predominance of non-monosomic GCs (>60% XX or XXX cells) across multiple follicles exhibited FD values above the average age-normalized reference level reported for healthy adolescents (9). Regardless of the peripheral blood karyotype, X-monosomic cells represented the predominant stromal cell population in all ovarian tissue samples analyzed, accounting for 69–89% of stromal cells (**Figures 5E–H**, **6**).

### The relationship between X chromosome mosaicism and ovarian reserve parameters

No clear relationship was observed between the proportion of Х-aneuploid cells in peripheral blood lymphocytes, ovarian stromal cells, and GCs. Similarly, no consistent monotonic relationship was apparent between OR markers (AMH, inhibin B), FD, and the proportion of X-monosomic cells in the analyzed cell populations. Given the exploratory nature of the study and the very limited sample size, these observations should be interpreted descriptively.

## Discussion

To our knowledge, this is the first study to provide a comprehensive evaluation of OR in adolescents with mosaic TS and spontaneous pubertal development. The assessment included hormonal and ultrasonographic markers of ovarian function, as well as morphological, morphometric and immunohistochemical, and molecular cytogenetic characterization of ovarian tissue. In addition, we explored the relationship between OR parameters and the proportion of X chromosome aneuploidy in peripheral blood lymphocytes and ovarian somatic cells.

Although all participants demonstrated ongoing spontaneous pubertal development and generally preserved gonadotropin and estradiol concentrations, several markers of OR were reduced. Compared with healthy adolescents, AMH levels, ovarian volumes, and AFC were lower, with large effect sizes. At the individual level, AMH concentrations below 1.1 ng/mL were observed in three patients and inhibin B concentrations below <10 pg/mL were detected in two of them. Nadesapillai et al. reported that follicle detection is associated with inhibin B concentrations of ≥10 ng/L (21). Our findings suggest that ovarian follicles may still be present in individuals with lower inhibin B concentrations, provided that AMH remains detectable (>0.1 ng/mL) and FSH concentrations remain within the normal range (<10 IU/L).

In our previous study, primordial follicles with pale or absent nuclei and contracted oocyte cytoplasm were observed more frequently in patients with TS than in individuals with normal female karyotypes. Nevertheless, no differences were found in the prevalence of follicles containing karyopyknotic GCs and/or oocytes (38). Consistent with previous reports by Mamsen et al. and Nadesapillai et al., we identified a substantial proportion of morphologically abnormal follicles showing signs of degeneration (10, 21). However, morphologically normal primordial and growing follicles were present in all ovarian tissue samples, suggesting that follicular development may proceed differently among individual follicles in adolescents with mosaic TS.

Previously we demonstrated that the expression of oocyte-specific markers, including BMP15 and CD117 in oocytes and GDF9 in GCs from patients with TS was broadly comparable to that observed in controls without chromosomal abnormalities. However, GDF9 expression within the ooplasm was lower in individuals with TS than in controls (38). Low cytoplasmic expression of GDF9 in oocytes is expected in resting primordial follicles because this growth factor plays a key role in granulosa-cell proliferation and theca-cell differentiation during follicular activation (39, 40). Compared with controls, reduced GDF9 expression in some participants may reflect differences in oocyte recruitment and maturation status (41). In animal models, expression of CD117, GDF9, and BMP15 decreases with reproductive aging and has been associated with oocyte developmental competence (42). The lower GDF9 expression observed in patients with TS was primarily attributable to a reduced proportion of positively stained oocytes rather than diminished staining intensity. Importantly, expression of CD117 and BMP15 remain detectable, suggesting that developmental pathways involved in follicular growth were preserved in at least some follicles (40, 41). Although participant P2 demonstrated weaker expression of GDF9 and BMP15 than the other participants, a broad spectrum of growing follicles was still present within the ovarian tissue.

Taken together, the preserved expression of CD117 together with reduced GDF9 and BMP15 expression in some oocytes may be consistent with differences in oocyte-granulosa cell signaling. Because GDF9 and BMP15 function as paracrine regulators of granulosa-cell proliferation, the possibility of altered cell-cell interactions associated with X chromosome aneuploidy cannot be excluded (43–46). However, direct functional studies are required to investigate this hypothesis.

Only a limited number of studies have examined X chromosome mosaicism in ovarian tissue from individuals with TS. Peek et al. reported that most GCs in small follicles from four of five patients contained a single X chromosome, whereas the majority of examined oocytes had a normal X chromosome complement. The proportion of monosomic and non-monosomic stromal cells was similar to that observed in peripheral blood lymphocytes in some patients but differed substantially in others. Importantly, no association was identified between FD and X chromosome mosaicism in ovarian somatic cells or oocytes (20). Similarly, Schleedoorn et al. and Nadesapillai et al. described normal X chromosomes complements in immature oocytes from two adolescents with X-monosomy in peripheral blood lymphocytes and GCs, while X-mosaicism was present in ovarian stromal cells (22, 24). Balen et al. also reported cytogenetically normal oocytes obtained after ovulation stimulation in a patient with a 45,X/46,XX mosaic karyotype and observed lower rates of X-monosomy in ovarian stromal cells than in peripheral blood lymphocytes (47).

Collectively, these studies suggest that oocytes in many individuals with TS retain normal X chromosome complement, whereas ovarian somatic cells frequently exhibit varying degrees of X chromosome mosaicism. Because follicular development depends on extensive communication between oocyte and GCs through gap junctions and paracrine signaling pathways, differences in the chromosomal composition of GCs may be associated with variation in follicular characteristics (11, 48). Furthermore, the prolonged cell cycle reported in 45,X cells could theoretically influence granulosa-cell proliferation during follicular growth, which normally involves multiple rounds of mitotic division (49, 50).

Particularly relevant are the findings of Peek et al., who transplanted ovarian cortical tissue from young patients with TS into severely immunodeficient mice (23). Prior to transplantation, most small follicles contained predominantly X-monosomic GCs, whereas the majority of oocytes exhibited a normal X chromosome complement. Following transplantation and hormonal stimulation, growing follicles developed in all analyzed samples. Interestingly, the proportion of X-aneuploid GCs in secondary and antral follicles was generally lower than that observed in small follicles before transplantation. In some cases, GCs carrying two X chromosomes became the predominant population in developing follicles. Moreover, in patients whose examined follicles initially contained exclusively X-monosomic GCs, X chromosome mosaicism was observed in growing follicles following tissue transplantation. The increased proportion of non-monosomic GCs during follicular growth may reflect the preferential proliferation of euploid cells during follicular maturation or the selective development of follicles containing a higher proportion of GCs with the normal sex chromosome complement (23).

Nadesapillai et al. subsequently demonstrated an increase in the number of primary and secondary follicles following *in vitro* growth (IVG) culture of ovarian cortical tissue from an adolescent with complete X-monosomy (24). Although abnormalities of the granulosa-cell layer and thickening of the basal lamina were observed, post-culture FISH analysis was not performed, precluding assessment of the relationship between these morphological findings and chromosomal composition (24).

Consistent with previous reports, all oocytes examined in our study demonstrated a normal X chromosome complement. Unlike earlier studies, however, complete X-monosomy was not observed in GCs from any participant. Instead, considerable variability in X chromosome mosaicism was observed both among follicles within the same ovary and relative to the level of X-aneuploidy in peripheral blood lymphocytes (**Figure 5**). The difference in the proportion of X-monosomic cells between peripheral blood lymphocytes and GCs ranged from 1% to 62%, whereas interfollicular variability within the same ovary ranged from 2% to 61%. In contrast, ovarian stromal cells consistently demonstrated high proportion of X chromosome aneuploidy (>69%) across all participants.

Although no statistical association could be demonstrated in this exploratory series, descriptive differences in FD were observed among participants with different proportions of non-monosomic GCs. Specifically, only participants whose follicles contained more than 60% non-monosomic GCs (XX or XXX) across multiple follicles exhibited FD values exceeding age-adjusted reference means. Considering the available evidence, it cannot be excluded that predominance of X-monosomic GCs is associated with differences in follicular characteristics; however, this observation requires further investigation. Notably, participant P5 had the highest proportion of non-monosomic lymphocytes in peripheral blood but demonstrated a predominance of X-aneuploid GCs and the lowest FD. At the same time, our histological and immunohistochemical findings, together with previous observations reported by Peek et al. indicate that follicular growth and development may remain preserved in adolescents with mosaic TS (23). These findings highlight the importance of comprehensive clinical, laboratory, and ultrasonographic assessment of OR when evaluating ovarian function in patients with TS, as the degree of X chromosome aneuploidy in peripheral blood lymphocytes does not necessarily reflect the proportion of X-monosomic GCs within the ovary.

The principal limitation of this exploratory study is the small sample size, which precludes definitive conclusions regarding the relationship between OR parameters and X chromosome aneuploidy in ovarian somatic cells. Furthermore, ovarian tissue X chromosome mosaicism cannot currently be considered a validated prognostic marker of fertility-preservation outcomes. Although a possible association between the chromosomal status of GCs and follicle-density patterns was observed, this finding should be interpreted cautiously and requires confirmation in larger prospective cohorts.

## Conclusion

In adolescents with mosaic TS and spontaneous pubertal development, we identified morphologically normal ovarian follicles expressing key growth factors and proteins involved in preantral folliculogenesis in most ovarian biopsy samples.

A high proportion of X-monosomic stromal cells and varying degrees of X chromosome mosaicism in GCs were observed in all examined ovarian cortical samples. No evidence of X chromosome aneuploidy was detected in oocytes. No clear relationship was observed between X-aneuploidy in peripheral blood lymphocytes and ovarian somatic cells or between OR markers, FD, and X chromosome mosaicism in blood and ovarian tissue. In this exploratory case series, age-appropriate FD was observed in participants who also demonstrated a predominance of non-monosomic GCs; however, no statistical association could be demonstrated.

Further studies are needed to clarify the relationship between ovarian-cell X chromosome mosaicism and follicular development in larger cohorts. Such studies may improve our understanding of the mechanisms underlying follicular loss and the potential role of ovarian tissue mosaicism in the variability of reproductive potential among individuals with TS.

## Data Availability

The raw data supporting the conclusions of this article will be made available by the authors, without undue reservation.
