# Supplementary material for: Ovarian function and X chromosome tissue mosaicism in adolescents with Turner syndrome and ongoing spontaneous puberty
Source: Front Endocrinol (Lausanne). 2026 Jul 7;17:1866671. doi: 10.3389/fendo.2026.1866671 (PMC13385041; doi:10.3389/fendo.2026.1866671)
Supplement: Supplementary file 1 [file SupplementaryFile1.docx]

SUPPLEMENTARY MATERIALS AND METHODS

The total number of follicles ($n_{follicles})$, ovarian tissue biopsy volume ($V)$, and follicle density ($FD, follicles/1 mm3$) were calculated using a correction factor (γ) to account for unexamined sections and a correction factor (α) to prevent repeated counting of the same follicle, according to the method described by Schmidt et al. (27):

$$n_{follicles}\text{= }\gamma\text{ * }\alpha\text{ * }\sum_{i\text{=}1}^{N} n_{i}$$

$$\text{ }V\text{= }\gamma\text{* }\sum_{i\text{=}1}^{N} A_{i}\text{*}t_{i}\text{ }$$

$$FD\text{= }\frac{n_{follicles}}{V}$$

*where* ***γ*** *= 5 because every fifth section was analysed; n_i_ represents the number of follicles counted in an individual section;* ***N*** *is the total number of analyzed sections;* ***A_i_*** *is the area of the section;* ***t_i_*** *is the section thickness.*

The avoid multiple counting of the same follicle, a modified correction factor (α) proposed by Hassan et al. (9) was applied:

$$\alpha\text{=}P_{q\text{+}1}\text{*}\frac{1}{q\text{+}1}\text{ + }P_{q\text{+}2}\text{*}\frac{1}{q\text{+}2}{,\text{ }\text{in}\text{ }which\text{ }P}_{q\text{+}1}\text{=}\frac{\left( q\text{+}1 \right)\text{ * }t\text{-}d}{t},\text{ }P_{q\text{+}2}\text{= }\frac{d\text{ - }(q\text{ * }t)}{t}$$

*Where* ***t*** *is the section thickness;* ***d*** *is the mean diameter of largest 10% of oocytes measured within a section;* ***q*** *is the quotient obtained by dividing follicle diameter by section thickness and rounding the result down to the nearest integer.*
